# The Role of *Piper sarmentosum* Aqueous Extract as a Bone Protective Agent, a Histomorphometric Study

**DOI:** 10.3390/ijms21207715

**Published:** 2020-10-19

**Authors:** Siti Fadziyah Mohamad Asri, Ima Nirwana Soelaiman, Mohamad Aris Mohd Moklas, Nurul Huda Mohd Nor, Nurul Hayati Mohamad Zainal, Elvy Suhana Mohd Ramli

**Affiliations:** 1 Department of Human Anatomy, Faculty of Medicine and Health Sciences, Universiti Putra Malaysia, UPM Serdang 43400, Malaysia; aris@upm.edu.my (M.A.M.M.); hudamohdnor@upm.edu.my (N.H.M.N.); mz_nurul@upm.edu.my (N.H.M.Z.); 2 Department of Pharmacology, Faculty of Medicines, Universiti Kebangsaan Malaysia Medical Centre (UKMMC), Jalan Yaacob Latif, Bandar Tun Razak, Kuala Lumpur 56000, Malaysia; imasoel@ppukm.ukm.edu.my; 3 Department of Anatomy, Faculty of Medicines, Universiti Kebangsaan Malaysia Medical Centre (UKMMC), Jalan Yaacob Latif, Bandar Tun Razak, Kuala Lumpur 56000, Malaysia

**Keywords:** glucocorticoid, histomorphometry, osteoporosis, *Piper sarmentosum*

## Abstract

Glucocorticoids are one of the causes of secondary osteoporosis. The aqueous extract of *Piper sarmentosum* contains flavonoids that possess antioxidant effects. In this study, we determined the effects of aqueous *Piper sarmentosum* leaf extract on structural, dynamic and static histomorphometric changes from osteoporotic bones of rats induced with glucocorticoids. Thirty-two Sprague-Dawley rats were divided equally into four groups—Sham control group given vehicles (intramuscular (IM) olive oil and oral normal saline); AC: Adrenalectomised (Adrx) control group given IM dexamethasone (DEX) (120 μg/kg/day) and vehicle (oral normal saline); AP: Adrx group administered IM DEX (120 μg/kg/day) and aqueous *Piper sarmentosum* leaf extract (125 mg/kg/day) orally; and AG: Adrx group administered IM DEX (120 μg/kg/day) and oral glycyrrhizic acid (GCA) (120 mg/kg/day). Histomorphometric measurements showed that the bone volume, trabecular thickness, trabecular number, osteoid and osteoblast surfaces, double-labelled trabecular surface, mineralizing surface and bone formation rate of rats given aqueous *Piper sarmentosum* leaf extract were significantly increased (*p* < 0.05), whereas the trabecular separation and osteoclast surface were significantly reduced (*p* < 0.05). This study suggests that aqueous *Piper sarmentosum* leaf extract was able to prevent bone loss in prolonged glucocorticoid therapy. Thus, *Piper sarmentosum* has the potential to be used as an alternative medicine against osteoporosis and osteoporotic fractures in patients undergoing long-term glucocorticoid therapy.

## 1. Introduction

Osteoporosis is a systemic skeletal disease involving low bone mass and microarchitectural deterioration of bone tissue, leading to high bone fragility and fracture risk [1]. Osteoporosis fracture is associated with significant morbidity and mortality.

Glucocorticoids are the main cause of secondary osteoporosis, which can occur at all ages. About 30–50% of patients receiving long-term glucocorticoid therapy are at risk of bone loss and fractures [2]. Risk factors associated with glucocorticoid-induced osteoporosis are aging, low body mass index, increased 11β-hydroxysteroid dehydrogenase (11β-HSD) enzyme expression, low bone mineral density, high doses of glucocorticoids, underlying chronic diseases such as rheumatoid arthritis, and glucocorticoid receptor genotypes. Progression of bone loss occurs even with very low doses of pharmacological glucocorticoids; depending on the dosage and duration of the therapy received [3]. These changes affect trabecular bone more than cortical bone, thus increasing the risk of fracture [4,5].

Glucocorticoids inhibit osteoblast function by reducing cell proliferation and differentiation; and inducing apoptosis of osteoblasts and mature osteocytes [6]. However, glucocorticoids cannot balance these effects by inhibiting bone resorption because glucocorticoid receptors have a reduced effect on mature osteoclasts [7]. Osteoclast activities are controlled indirectly by factors triggered from osteoblasts. The important stage in osteoclast differentiation is expression of receptor activator NF-ĸB (RANK) on the surface of the osteoblast by RANK ligand (RANKL), and inhibition by osteoprotegerin (OPG), which is also secreted by osteoblasts. Glucocorticoids can affect the ratio of RANKL/OPG by acting upon these two factors, resulting in osteoclastogenesis, thus speeding up the early phase of bone resorption.

Both isoenzymes of 11β-hydroxysteroid dehydrogenase are important in the regulation of corticosteroid action. They catalyze the interconversion of active cortisol to inactive cortisone in humans, and active corticosterone to inactive 11-dehydrocorticosterone in rodents through pre-receptor action [8,9,10]. 11β-HSD type 1, a low-affinity NADPH-dependent bidirectional enzyme, predominantly reductase, can interconvert cortisone to cortisol. Whereas 11β-HSD type 2, a high-affinity NAD-dependent unidirectional enzyme, deactivates cortisol to cortisone with dehydrogenase action. 11β-HSD type 1 reductase activity is responsible for the local action of glucocorticoids in bone, expressed in osteoblasts and osteoclasts. Eijken et al. [11] reported a converse relationship between osteoblast differentiation and 11β-HSD1 enzyme activity after long-term treatment with dexamethasone.

Liquorice, the dried and processed root from the herbal plant *Glycyrrhiza glabra* originating from East Asia up to the Mediterranean, had been widely used in traditional medicine for treating stomach ulcer, cough, arthritis, autoimmune diseases and adrenal insufficiency. An active component of liquorice roots, glycyrrhizic acid (GCA), is converted into glycyrrytinic acid (GRA) in the body and is responsible for most of the effects of liquorice [12]. The chemical structure of GCA and GRA is the same as the hormone aldosterone, deoxycorticosterone and cyclopentanophenanthrene steroids [13], thus it can amend the activation of the glucocorticoid receptors (GR) [14]. Carbenoxolone is one of the synthetic drugs from glycyrrytinic acid that inhibits 11β-HSD1 enzyme activity in the liver, thereby reducing lipids in the blood [15]. Glycyrrhizic acid is a potent inhibitor of 11β-HSD1, which is used to monitor the procurement of cortisol in the liver, fatty tissues and bones [16]. The reduction of local turnover of cortisol by liquorice can have an advantageous effect by preventing the effects of osteoporosis caused by excessive glucocorticoids (GC). Short-term glucocorticoid therapy does not affect the level of piridinoline and deoxypiridinoline, but the bone resorption level can be reduced by preventing the activities of 11β-HSD1 in osteoclast cells [17].

*Piper sarmentosum* (Piperaceae) is a type of herbal plant commonly consumed as traditional cuisine [18,19,20]. According to Malay tradition, it is used for treating headache, toothache, cough, asthma and fever. It is known locally in Malaysia as ‘daun kaduk’. Phytochemical studies of *Piper* species led to the separation of several clusters of active components, such as amides, propenylphenols, lignans, steroids, flavonoids, ketones, carboxylic acids, phytosterols and phenyls [21,22,23]. Many studies have shown that *Piper sarmentosum* possesses pharmacological effects such as anti-tuberculosis [19,24], anti-cancer [25], hypoglycaemic action [26,27], anti-malarial [28], antioxidant [29], neuromuscular blocking action [30], anti-atherosclerosis [31] and anti-inflammation [32] activities. Our previous study on *Piper sarmentosum* aqueous extract at 125 mg/kg/day [33] showed that it promotes fracture healing in osteoporotic bone. Ramli et al. [34] studied the effects of the same dose of aqueous *Piper sarmentosum* leaf extract on bone remodelling and noted that the expression of 11β-HSD type 1 enzyme was reduced and the dehydrogenase activity of the enzyme was increased in the femurs of adrenalectomised rats treated with long-term dexamethasone.

Dexamethasone is a synthetic steroid that is approximately 20–30 times more potent than hydrocortisone and 4–5 times more potent than prednisolone. One previous study showed that dexamethasone-induced osteoporosis was caused by inhibition of osteoblast proliferation and induction of mature osteoblast apoptosis [35]. Dexamethasone also resulted in an increased number of osteoclasts, consequently improving bone resorption [36]. Endogenous and exogenous glucocorticoids showed the same supraphysiologic actions on bones [37].

Bone remodeling occurs in two distinct phases—resorption of the existing mineralized bone matrix by osteoclasts followed by formation of new bone by osteoblasts [38]. Bone histomorphometry is a method used to analyze the changes that occur in the bone structure on three levels—microstructure, cellular and growth. Bone histomorphometry is very useful for identifying and describing pathogenesis and cellular mechanisms that occur in various diseases related to bone metabolism, such as glucocorticoid-induced osteoporosis [39]. The structural and static histomorphometry parameters measure the microstructure and cellular components of the trabecular bone respectively, whereas the dynamic histomorphometry parameters reflect the growth of the bone. The double-labelled surface of the bone (dLS/BS) refers to the on-going bone formation process, whereas the single labelled surface bone (sLS/BS) refers to the beginning and ending of the bone formation. The mineralized surface (MS/BS) is the percentage of active mineralization on bone surfaces. It is calculated by adding the extent of dLS/BS with half the extent of the sLS/BS. The MS/BS reflects the overall function of the bone surface, where new mineralized bone is deposited within the period of calcine labelling. The mineralized apposition rate (MAR) is a linear measurement of new bone deposition. It is calculated as the mean distance between the extents of dLS/BS divided by the time periods of calcine labelling. The bone formation rate (BFR/BS) is calculated by multiplying the MS/BS with the MAR. Dynamic histomorphometry parameters were used to assess bone formation activity. Bone resorption activity was assessed by the static histomorphometry parameter, osteoclast surface per bone surface (OcS/BS) [40].

The aim of this study was to evaluate the protective effects of the aqueous extracts of *Piper sarmentosum* leaves on bone remodelling through histomorphometric analysis. The results of this study may provide an important resource for improving the bone strength of patients on prolonged glucocorticoid therapy.

## 2. Materials and Methods

### 2.1. Preparation of Aqueous Piper sarmentosum Leaf Extract

*Piper sarmentosum* was recognized by the Biology Herbarium Unit, Faculty of Environmental Science and Natural Resources Sciences, Universiti Kebangsaan Malaysia. A verification voucher, UKMB 29,851 of the specimens was obtained. *Piper sarmentosum* leaves were dried using an oven under constant airflow, then grounded and boiled with distilled water. The aqueous extract solution was sent to Forest Research Institute of Malaysia (FRIM) for freeze-drying to obtain the powder form of *Piper sarmentosum*.

### 2.2. Animals and Treatment

All procedures followed the guidelines of the Universiti Kebangsaan Malaysia (UKM) Research and Animal Ethics Committee (UKMAEC) (No: PP/ANT/2010/ELVY/14-JULY/313-JULY-2012-MAY-2012; 11 August 2010) for ethical handling of laboratory animals. Thirty-two male Sprague-Dawley rats, weighing 250–300 g, were purchased from the UKM Animal Breeding Centre. The rats were divided into four groups of eight—Sham control rats given vehicle intramuscular (IM) olive oil (0.05 mL/kg) and normal saline (1 mL/kg) orally as vehicle; AC: Adrenalectomised (Adrx) control rats given IM dexamethasone (DEX) (120 μg/kg/day) and normal saline (1 mL/kg) orally as vehicle; AP: Adrx rats given IM DEX (120 μg/kg/day) and aqueous *Piper sarmentosum* (*Ps*) leaf extract (125 mg/kg/day) orally, and AG: Adrx rats given IM DEX (120 μg/kg/day) and glycyrrhizic acid (GCA) (120 mg/kg/day) via oral gavage [34].

Two weeks after receiving the rats, adrenalectomy were done. The rats were given Ketapex (5 mg/100 g body weight) and Xylazil (1 mg/100 g body weight) as anaesthetic drugs. Dorsal midline and bilateral flank muscle incisions were carefully made, then the adrenal glands were identified and removed. The incisions were sutured and Poviderm cream was applied to the wound daily as a prevention for infection, and to assist with wound healing. The rats were given IM injections of Baytril 5% post-adrenalectomy for 5 days as prophylaxis. The sham control rats had a similar procedure except that the adrenal glands were left in-situ. Treatment was started after two weeks post-adrenalectomy. Dexamethasone [41,42] was dissolved in olive oil and given intramuscularly (120 μg/kg/day) for 6 days a week [43]. *Piper sarmentosum* of 125 mg/kg/day, and GCA of 120 µg/kg/day were diluted in normal saline and given orally via oral gavage for 8 weeks [27,44,45]. The sham control rats were given the same volumes of vehicle (olive oil intramuscularly, and normal saline orally). The dexamethasone control rats (AC) were also given normal saline orally as vehicle. The treatment was given for 6 days a week for 8 consecutive weeks. The rats were kept in cages under natural sunlight during the day, and darkness at night. Pellets ad libitum were given to the rats. Tap water ad libitum was given to the sham control rats to drink, while the adrenalectomised rats were given normal saline [41]. After 8 weeks of treatment, the rats were sacrificed humanely, and the left femurs were taken for bone histomorphometric analysis.

### 2.3. Bone Histomorphometric Analysis

Histomorphometric analysis was done using a combination of manual and automated techniques with appropriate computer software. The selected areas were from the metaphyseal part of the left distal femoral bone. The distal part was then cut into two for decalcified and undecalcified preparations. For the decalcification process, the bony part was mixed with EDTA solution until it became softer, and then stained with Masson–Goldner Trichrome. The undecalcified part was molded using Osteo-Bed resin solutions and stained with von Kossa. The metaphysis (metaphyseal part) is located between 3 mm to 7 mm from the lowest point of the growth plate line, and 1 mm from the lateral cortex [46]. This part was chosen because the bone mass of this part decreases as a result of trabecular bone retardation during the process of bone remodelling [47]. Structural, static and dynamic histomorphometric measurements were carried out to assess the impact of treatment in relation to the microstructure, formation and resorption of the bone [39]. These values are based on a formula proposed by the Committee of Histomorphometry Nomenclature ASMBR [48]. Each sample of bone was sectioned to produce eight slides. For each slide, three metaphyseal areas were selected for observation. The average of these three areas was taken as a value for one sample. The average of eight slides was taken as a value for one group.

#### 2.3.1. Bone Microstructure Histomorphometric Analysis

**Figure 1:**
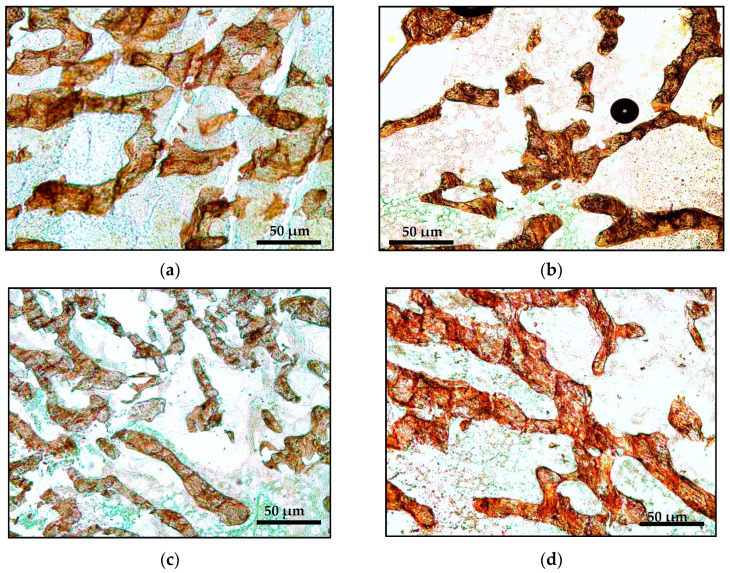
Photomicrograph of decalcified trabecular bones for structural histomorphometry parameters using von Kossa staining (50X magnification): (**a**) Sham; (**b**) AC: Adrx control given IM DEX 120 μg/kg/day; (**c**) AP: Adrx rats given IM DEX 120 μg/kg/day and aqueous *Piper sarmentosum* extract 125 mg/kg/day; and (**d**) AG: Adrx rats given IM DEX 120 μg/kg/day and GCA 120 mg/kg/day.

The structural histomorphometry parameters were measured automatically using Video Software-Test Master 4.0. This measurement was done using undecalcified bone samples stained with von Kossa (Figure 1). The parameters obtained were bone volume/tissue volume (BV/TV), trabecular thickness (Tb.Th), trabecular number (Tb.N) and trabecular separation (Tb.S). The software provides an automatic calculation of bone volume (BV), bone surface (BS) and tissue volume (TV). Tb.Th were calculated by BV/(BS × 0.5). Tb.N were calculated by (BV/TV)/Tb.Th. Tb.Sp were calculated by (1/Tb.N) − Tb.Th.

#### 2.3.2. Bone Static Histomorphometric Analysis

**Figure 2:**
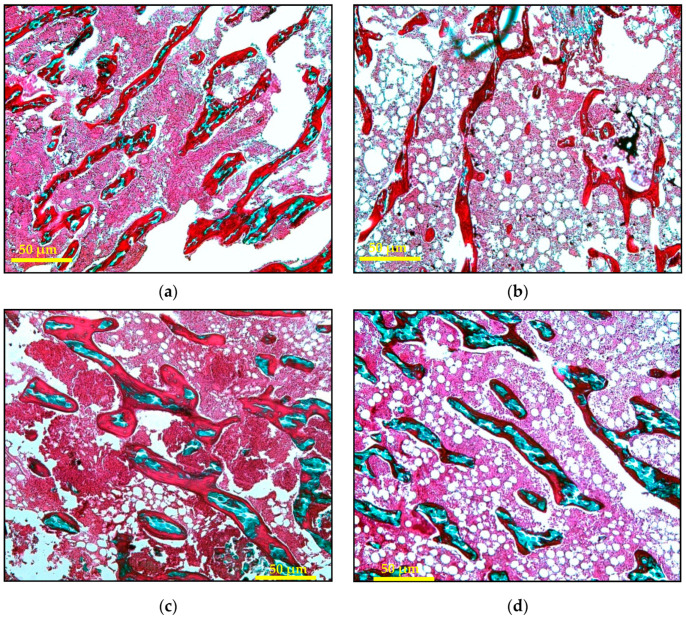
Photomicrograph of decalcified trabecular bones for static histomorphometry parameters using Masson–Goldner Trichrome staining (50X magnification): (**a**) Sham; (**b**) AC: Adrx control given IM DEX 120 μg/kg/day; (**c**) AP: Adrx rats given IM DEX 120 μg/kg/day and aqueous *Piper sarmentosum* extract 125 mg/kg/day; and (**d**) AG: Adrx rats given IM DEX 120 μg/kg/day and GCA 120 mg/kg/day.

The static histomorphometry parameters were performed on decalcified bone samples stained with Masson–Goldner Trichrome showing green coloured mineralised bone and dark red coloured osteoid (Figure 2). Osteoblast and osteoclast cells could be clearly seen. Osteoblast cells have a single nucleus with a cytoplasm rich in organelles. They form a line on the surface of the osteoid. Osteoclast cells are multinucleated large cells with eosinophilic cytoplasm that are found close to the Howship’s lacunae. The parameters obtained were trabecular bone volume (BV), trabecular bone surface (BS), osteoid volume (OV/BV), osteoid surface (OS/BS), osteoblast surface (Ob.S), and osteoclast surface (Oc/S). The calculation of these parameters was done manually based on the modified Weibel technique [49].

#### 2.3.3. Bone Dynamic Histomorphometric Analysis

**Figure 3:**
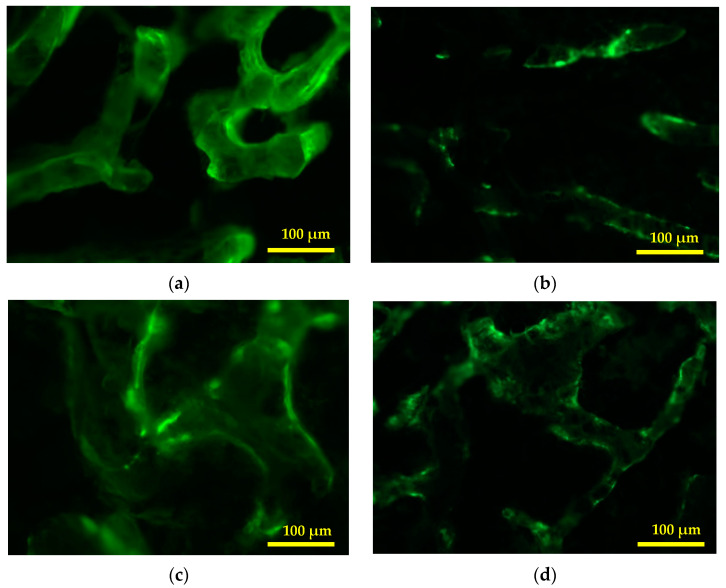
Photomicrograph of undecalcified trabecular bones captured with a fluorescent microscope and displayed on a computer with Image Pro-Express software for dynamic histomorphometry parameters (100X magnification): (**a**) Sham; (**b**) AC: Adrx control given IM DEX 120 μg/kg/day; (**c**) AP: Adrx rats given IM DEX 120 μg/kg/day and aqueous *Piper sarmentosum* extract 125 mg/kg/day; and (**d**) AG: Adrx rats given IM DEX 120 μg/kg/day and GCA 120 mg/kg/day.

The dynamic histomorphometric measurements were performed on undecalcified bone without staining. The images produced were connected to a fluorescent microscope and displayed on a computer with Image Pro-Express software (Figure 3). The parameters obtained were trabecular surface (BS), single-labelled trabecular surface (sLS/BS), double-labelled trabecular surface (dLS/BS), mineralised surface (MS/BS), mineralised apposition rate (MAR), and bone formation rate (BFR/BS). The software provides an automatic calculation of BS, sLS and dLS. MS/BS were calculated by equation of ((dLS + (sLS × 0.5))/BS. MAR were calculated by dividing the width between two fluorescent labels with days of injecting calcein. BFR/BS were calculated by (MS/BS) × MAR.

### 2.4. Statistical Analysis

The data were statistically analysed using the software Statistical Package for Social Sciences (SPSS) version 25. The Shapiro–Wilk normality test was used to determine the distribution of the data as the sample size was less than 100. All of the groups were found to be normally distributed. Parametric statistics (ANOVA test) and Tukey’s post-hoc test were used for comparison between treatment groups. Data were expressed as mean ± standard error of the mean (SEM) (Table 1, Table 2 and Table 3). The level of significance was set at *p* < 0.05.

## 3. Results

### 3.1. Structural Histomorphometry Parameters

**Figure 4:**
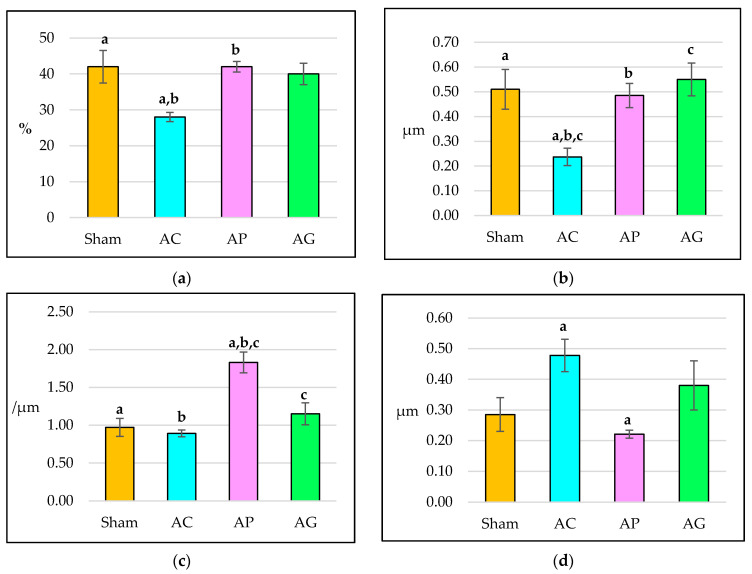
Structural trabecular histomorphometry parameters after 8 weeks of treatment: (**a**) Bone volume (BV/TV); (**b**) Trabecular thickness (Tb.Th); (**c**) Trabecular number (Tb.N); (**d**) Trabecular separation (Tb.Sp). AC: Adrx control given IM DEX 120 μg/kg/day; AP: Adrx rats given IM DEX 120 μg/kg/day and aqueous *Piper sarmentosum* extract 125 mg/kg/day; and AG: Adrx rats given IM DEX 120 μg/kg/day and GCA 120 mg/kg/day. Data presented as mean ± SEM. Same letters indicate significant difference between groups at *p* < 0.05.

Dexamethasone treatment for eight weeks significantly reduced the bone volume of AC compared to Sham. BV/TV was significantly higher in AP compared to AC. AP and AG were not significantly differ from Sham (Figure 4a).

Femoral trabecular thickness of AC was significantly lower compared to Sham after eight weeks of treatment. Tb.Th of AP and AG were significantly higher compared with AC. AP and AG did not differ significantly from the Sham (Figure 4b).

The trabecular number of AC did not differ significantly compared with the Sham after eight weeks of treatment. The trabecular number of AP was significantly higher compared to other groups after supplementation with aqueous *Piper sarmentosum* leaf extract. There was no significant difference in Tb.N of AG compared to Sham (Figure 4c).

Rats that received DEX treatment in AC for eight weeks did not show significant differences in Tb.Sp compared to Sham. Supplementation of aqueous *Piper sarmentosum* leaf extract in AP reduced the Tb.Sp significantly compared to AC. AP and AG did not differ significantly from Sham (Figure 4d).

### 3.2. Static Histomorphometry Parameters

**Figure 5:**
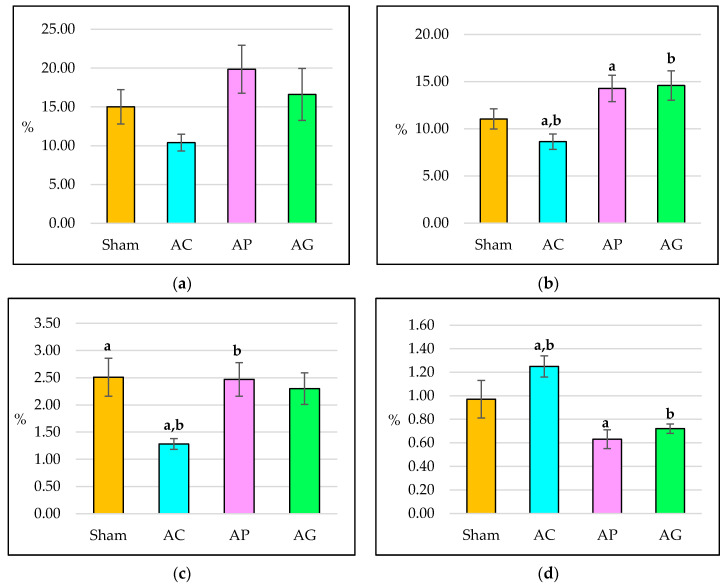
Static trabecular histomorphometry parameters after 8 weeks of treatment: (**a**) Osteoid volume (OV/BV); (**b**) Osteoid surface (OS/BS); (**c**) Osteoblast surface (Ob.S); (**d**) Osteoclast surface (Oc.S). AC: Adrx control given IM DEX 120 μg/kg/day; AP: Adrx rats given IM DEX 120 μg/kg/day and aqueous *Piper sarmentosum* extract 125 mg/kg/day; and AG: Adrx rats given IM DEX 120 μg/kg/day and GCA 120 mg/kg/day. Data presented as mean ± SEM. The same letters indicate significant differences between groups at *p* < 0.05.

Dexamethasone treatment for eight weeks did not produce significant differences to the OV/BV in all groups (Figure 5a).

Trabecular osteoid surface in AC was not significantly different compared with Sham after eight weeks of treatment. Supplementation of aqueous *Piper sarmentosum* leaf extract and GCA in AP and AG respectively enhanced the OS/BS significantly compared with AC. AP and AG did not differ significantly from the Sham (Figure 5b).

Dexamethasone treatment for eight weeks resulted in a significant decrement of Ob.S in AC compared to Sham. Supplementation of aqueous *Piper sarmentosum* leaf extract in AP significantly increased the Ob.S compared with AC. AP and AG did not differ significantly from the Sham (Figure 5c).

Dexamethasone treatment for eight weeks significantly increased the Oc.S in trabecular bone of AC compared to AP and AG. AP and AG did not differ significantly from the Sham (Figure 5d).

### 3.3. Dynamic Histomorphometry Parameters

**Figure 6:**
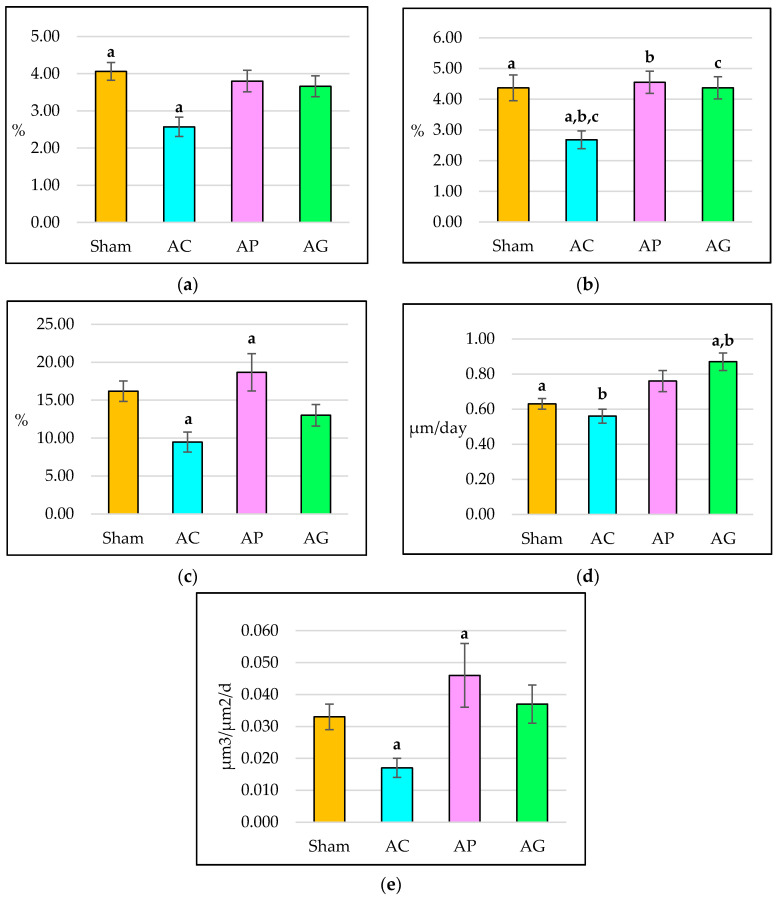
Dynamic trabecular histomorphometry parameters after 8 weeks of treatment: (**a**) Single-labelled trabecular surface (sLS/BS); (**b**) Double-labelled trabecular surface (dLS/BS); (**c**) Mineralized surface (MS/BS); (**d**) Mineral apposition rate (MAR); (**e**) Bone formation rate (BFR/BS). AC: Adrx control given IM DEX 120 μg/kg/day; AP: Adrx rats given IM DEX 120 μg/kg/day and aqueous *Piper sarmentosum* extract 125 mg/kg/day; and AG: Adrx rats given IM DEX 120 μg/kg/day and GCA 120 mg/kg/day. Data presented as mean ± SEM. The same letters indicate significant differences between groups at *p* < 0.05.

Rats that received DEX treatment for eight weeks showed a significant decrease in sLS/BS in AC compared with Sham. AP and AG respectively did not show a significant difference compared to AC. AP and AG did not differ significantly from Sham (Figure 6a).

The double-labelled trabecular surface in AC was significantly lower than Sham. AP and AG showed significantly higher dLS/BS compared to AC. AP and AG did not differ significantly from Sham (Figure 6b).

The mineralized surface in the AC was not significantly different compared to the Sham. Supplementation of aqueous *Piper sarmentosum* leaf extract in the AP increased the MS/BS significantly compared to the AC. AP and AG did not differ significantly from the Sham (Figure 6c).

The mineral apposition rate in the AC was not significantly different compared to the Sham. MAR in the AG was significantly higher compared with Sham and AC. AP did not differ significantly compared to Sham and AC (Figure 6d).

Bone formation rate in AC was not significantly different compared with Sham. Supplementation of aqueous *Piper sarmentosum* leaf extract in AP increased the BFR/BS significantly compared to AC. AP and AG did not differ significantly from Sham (Figure 6e).

## 4. Discussion

In this study, adrenalectomised rats were injected with dexamethasone and were used as a glucocorticoid-induced osteoporotic model. The dosage and length of time for dexamethasone treatment was based on a study by Elvy Suhana et al. [44]. Previous studies have found that dexamethasone treatment for two months decreased the calcium content and BMD of adrenalectomised rats [41,49]. The rats in this study underwent adrenalectomy to prevent the effects of endogenous glucocorticoid that will be influenced by circadian rhythm, as well as physical and emotional stress. It was replaced with an exogenous glucocorticoid, dexamethasone, with constant dosage to ensure homogenous glucocorticoid level in the rats’ body. Thus, the appropriate dosage required to induce osteoporosis can be determined without interference from endogenous glucocorticoids. The absence of adrenal suppression will cause these rats to lose mineralocorticoid hormones, but Ima Nirwana and Fakhrurazi [41] showed that the absence of mineralocorticoid does not affect the bone metabolism. The absence of mineralocorticoid will result in hypoaldosteronaemia, whereby the kidneys would be unable to absorb the sodium and chloride ions. Therefore, the adrenalectomised rats were subjected to oral intake of normal saline for sodium and chloride replacement.

Structural histomorphometric analysis showed that the bone volume (BV/TV) and trabecular thickness (Tb.Th) were reduced in rats receiving long-term treatment of dexamethasone, even though no changes in trabecular number (Tb.N) and trabecular separation (Tb.Sp) were observed. Changes in the bone microstructure were due to changes in bone remodelling involving dynamic and cellular parameters due to prolonged dexamethasone treatment. Static histomorphometric analysis showed reduction in bone formation indicated by reduced osteoblast surface (Ob.S). However, long-term dexamethasone treatment does not produce significant changes to osteoclast surface (Oc.S), osteoid surface (OS/BS), and osteoid volume (OV/BV). Dynamic histomorphometric analysis of rats receiving long-term dexamethasone treatment showed disturbances in bone formation and mineralisation, in which the single-labelled trabecular surface (sLS/BS) and double-labelled trabecular surface (dLS/BS) significantly declined. However, no significant changes were noted in the mineralised surface (MS/BS), mineral apposition rate (MAR) and bone formation rate (BFR/BS) in long-term dexamethasone-treated rats.

A study showed that dexamethasone treatment in healthy Sprague-Dawley rats significantly decreased the BV/TV and Tb.N, and increased the Tb.Sp and Oc.S significantly. Dexamethasone also significantly decreased the MS/BS, MAR and BFR/BS. Thus, this shows that dexamethasone inhibited bone formation and increased bone resorption, leading to osteopenia [50]. In our study, there were increments in the BV/TV, Tb.Th, Ob.S, sLS/BS and dLS/BS but no changes in the MS/BS, MAR and BFR; and the Oc.S. There were gradual changes in bone formation, whereby bone formation occurred in discrete basic multicellular units (BMUs), which are not continuously active. The formation period is the time when the bone surface is actively formed. Otherwise, the bone surface is either resorbing or quiescent for the rest of the time. The activation frequency of this formation period is related to the BFR; it depends on the amount of bone formed during each remodelling cycle, which is represented by the trabecular thickness (Tb.Th) [38]. This study showed increasing Tb.Th, hence the increasing BV/TV even though no changes were seen in the BFR/BS. The time interval between administrations of calcine labelling also affected the bone formation. Between the first and the last calcine labels, some BMUs stopped forming bone, while others just started to form bone, thus those BMUs will have a single label of calcien [38]. Our results showed significant increment in the sLS/BS and dLS/BS but no changes were found in the MS/BS, MAR and BFR. Therefore, we need a larger surface to measure the labelling. Other parameters that relate to the bone formation during bone turnover are Ob.S, OS/BS and the number of active osteoblasts. However, none of the parameters gave information as accurate as the calcine labelling [38]. Our study showed increasing Ob.S but no changes in OS/BS, possibly due to a reduced formation period.

Direct effects of glucocorticoids in bone remodelling involved coordination of osteoblasts during bone formation and osteoclasts during bone resorption [51]. Histomorphometric studies showed long-term dexamethasone treatment reduces the Ob.S, thus leading to a reduction in the sLS/BS and dLS/BS. This study is supported by a previous study indicating that 11β-HSD1 enzyme reductase activity increased, while osteoblast differentiation activity reduced [11,52]. Osteoblastogenesis was inhibited by long-term glucocorticoid action, while apoptosis of osteoblasts and osteocytes were enhanced [53]. The reduction in osteoblastogenesis by long-term dexamethasone treatment increased osteoblast apoptosis and reduced the BV/TV and Tb.Th. Reduced osteoblast function resulted in reduction of the single-labelled surface (sLS/BS) and double-labelled surface (dLS/BS) of osteoid formation. This coincides with the previous histomorphometric studies showing that long-term glucocorticoid treatments reduced osteoblasts, and reduced the trabecular bone volume and thickness [2,39].

*Piper sarmentosum* (*Ps*) is a herbal plant found in Southeast Asia. In Malaysia and Indonesia, this plant is used in traditional medicine for treating toothache, pleuritis, asthma, diabetes, hypertension, joint pain and fungal infections. Phytochemicals of *Ps* indicate that the methanol extracts of this plant contain naringenin, a flavonoid [23,29,54]. Flavonoids are known to reduce bone loss and increase bone strength in ovariectomised rats [55]. The preventive action of flavonoids in oxidative stress plays a role in reducing the risk of osteoporosis, and enhancing bone fracture recovery [56,57]. Recent studies showed a high antioxidative activity of flavonoids, even in the boiled plants [58]. The aqueous extract of *Ps* leaves contains the flavonoids rutin and vitexin, which have protective effects on cells against hydrogen peroxide [59]. Rutin increases osteoblast differentiation by increasing the OPG expression and this helps to increase bone formation [60,61]. We compared the effects of aqueous *Ps* leaf extract supplementation with those of glycyrrhizic acid (GCA) in dexamethasone-treated rats. Glycyrrhizic acid is a bioactive component in liquorice. The extract from liquorice has long been used in Asia as a traditional treatment for cough and other respiratory problems [62]. Glycyrrhizic acid is known as a strong inhibitor of the 11β-HSD1 enzyme, with the capability to reduce local corticosterone action. Subsequently, GCA was able to prevent the effects of osteoporosis caused by excess glucocorticoids [63]. Our previous studies concluded that GCA reduced the plasma corticosterone level in rats receiving continuous dexamethasone treatment [17]. Mohamad Asri et al. [64] showed that *Ps* had the ability to increase the dehydrogenase activity of the 11β-HSD1 enzyme, thus showing the same capability to reduce the action of corticosterone.

Histomorphometric study showed an increment in osteoblast surface (Ob.S) and osteoid surface (OS/BS), while the osteoclast surface (Oc.S) reduced in trabecular bones of rats receiving aqueous *Ps* leaf extract supplementation. *Piper sarmentosum* treatment also showed an increment in mineralised surface (MS/BS) caused by improvement in OS/BS and double-labelled surface (dLS/BS), and hence, increasing bone formation rate (BFR). *Piper sarmentosum* leaf extract, which contained the flavonoid, rutin, [65] was able to increase osteoblast differentiation by increasing OPG expression, thereby increasing bone formation [33,66]. The changes caused by aqueous *Ps* leaf extract to bone remodelling impacted the trabecular bone microstructures. This condition affects the bone mass, which contributes to bone strength. The bone microstructure study showed that trabecular number (Tb.N), trabecular thickness (Tb.Th) and trabecular bone volume (BV/TV) increased, while trabecular separation (Tb.Sp) reduced. We studied the comparison of the effects of aqueous *Ps* leaf extract supplementation with those of glycyrrhizic acid (GCA). The histomorphometric study in adrenalectomised rats given GCA showed that osteoclast surface (Oc.S) reduced significantly. The osteoid surface (OS/BS), osteoblast surface (Ob.S) and double-labelled surface (dLS/BS) increased, possibly due to increased osteoblast activities based on contemporaneous increase in the mineral apposition rate (MAR) [65]. These changes lead to an increase in the trabecular thickness (Tb.Th), even though the trabecular number (Tb.N) was reduced. GCA caused changes to bone mass and microstructure of trabecular bone in dexamethasone-induced osteoporotic rats.

Our previous biomechanical study of trabecular bone showed that the effects of aqueous *Ps* leaf extract and those of GCA were similar, whereby the bone strength and rigidity increased significantly [65]. Aqueous *Ps* leaf extract in rats receiving long-term dexamethasone treatment made the bone stronger by being able to withstand higher loads. These changes most likely contributed to maintaining bone biomechanical strength [65]. The use of *Ps* in rats undergoing ovariectomy also helped the recovery process after bone fracture [33,66] and maintained the bone biomechanical strength during fracture healing [67]. Maizura et al. [68] found that oral ingestion of aqueous *Ps* leaf extract up to 2000 mg/kg per day was not toxic to glucocorticoid-induced osteoporotic rodents and it also did not contribute to oxidative stress. *Piper sarmentosum* is also shown to have antioxidative effects [29,69], which may possibly contribute to its bone protective effects.

## 5. Conclusions

Aqueous *Piper sarmentosum* (*Ps*) leaf extract may improve the microstructure and cellular components of glucocorticoid-induced osteoporotic bones in rats, and hence, improve the growth of the bone. *Ps* might be able to protect the bones from negative effects of long-term glucocorticoid therapy, and thus, may have the potential to be used as a bone protective agent. Further study needs to be carried out to explore the mechanism(s) involved in the active compound(s) found in *Ps* in detail.

## Figures and Tables

**Table 1:** Mean values, standard error of mean (SEM), and standard deviation (SD) of structural histomorphometric parameters.

Parameter	Sham		AC		AP		AG	
	Mean ± SEM	SD	Mean ± SEM	SD	Mean ± SEM	SD	Mean ± SEM	SD
BV/TV (%)	42 ± 4.53	11.09	28 ± 1.29	3.16	42 ± 1.49	3.66	40 ± 2.97	7.27
Tb.Th (μm)	0.51 ± 0.08	0.20	0.24 ± 0.04	0.09	0.49 ± 0.05	0.12	0.55 ± 0.07	0.16
Tb.N (/μm)	0.97 ± 0.12	0.18	0.89 ± 0.05	0.12	1.83 ± 0.14	0.21	1.15 ± 0.15	0.22
Tb.Sp (μm)	0.29 ± 0.06	0.13	0.48 ± 0.05	0.13	0.22 ± 0.01	0.03	0.38 ± 0.08	0.20

BV/TV: bone volume/tissue volume; Tb.Th: trabecular thickness; Tb.N: trabecular number; Tb.Sp: trabecular separation. AC: Adrx control given IM DEX 120 μg/kg/day; AP: Adrx rats given IM DEX 120 μg/kg/day and aqueous *Piper sarmentosum* extract 125 mg/kg/day; and AG: Adrx rats given IM DEX 120 μg/kg/day and GCA 120 mg/kg/day.

**Table 2:** Mean values, standard error of mean (SEM), and standard deviation (SD) of static histomorphometric parameters.

Parameter	Sham		AC		AP		AG	
	Mean ± SEM	SD	Mean ± SEM	SD	Mean ± SEM	SD	Mean ± SEM	SD
OV/BV (%)	15.01 ± 2.21	5.42	10.40 ± 1.08	2.65	19.85 ± 3.10	7.59	16.60 ± 3.35	8.21
OS/BS (%)	11.04 ± 1.08	2.64	8.63 ± 0.82	2.00	14.27 ± 1.39	3.41	14.58 ± 1.55	3.80
Ob.S (%)	2.51 ± 0.35	0.86	1.28 ± 0.10	0.25	2.47 ± 0.31	0.76	2.30 ± 0.29	0.71
Oc.S (%)	0.97 ± 0.16	0.39	1.25 ± 0.09	0.23	0.63 ± 0.08	0.19	0.72 ± 0.04	0.09

OV/BV: osteoid volume/bone volume; OS/BS: osteoid surface/bone surface; Ob.S: osteoblast surface; Oc.S: osteoclast surface. AC: Adrx control given IM DEX 120 μg/kg/day; AP: Adrx rats given IM DEX 120 μg/kg/day and aqueous *Piper sarmentosum* extract 125 mg/kg/day; and AG: Adrx rats given IM DEX 120 μg/kg/day and GCA 120 mg/kg/day.

**Table 3:** Mean values, standard error of mean (SEM), and standard deviation (SD) of dynamic histomorphometric parameters.

Parameter	Sham		AC		AP		AG	
	Mean ± SEM	SD	Mean ± SEM	SD	Mean ± SEM	SD	Mean ± SEM	SD
sLS/BS (%)	4.06 ± 0.24	0.59	2.57 ± 0.26	0.64	3.80 ± 0.29	0.70	3.66 ± 0.28	0.69
dLS/BS (%)	4.37 ± 0.42	1.04	2.68 ± 0.29	0.70	4.55 ± 0.36	0.87	4.37 ± 0.36	0.87
MS/BS (%)	16.19 ± 1.34	3.29	9.47 ± 1.32	3.22	18.67 ± 2.46	6.01	13.01 ± 1.43	3.49
MAR (μm/day)	0.63 ± 0.03	0.01	0.56 ± 0.04	0.01	0.76 ± 0.06	0.01	0.87 ± 0.05	0.01
BFR/BS (µm^3^/µm^2^/d)	0.03 ± 0.004	0.01	0.02 ± 0.003	0.01	0.05 ± 0.010	0.02	0.04 ± 0.006	0.01

sLS/BS: single-labelled trabecular surface; dLS/BS: double-labelled trabecular surface; MS/BS: mineralizing surface/bone surface; MAR: mineral apposition rate; BFR/BS: bone formation rate/bone surface. AC: Adrx control given IM DEX 120 μg/kg/day; AP: Adrx rats given IM DEX 120 μg/kg/day and aqueous *Piper sarmentosum* extract 125 mg/kg/day; and AG: Adrx rats given IM DEX 120 μg/kg/day and GCA 120 mg/kg/day.
